# Role of TLR1, TLR2 and TLR6 in the modulation of intestinal inflammation and *Candida albicans* elimination

**DOI:** 10.1186/s13099-017-0158-0

**Published:** 2017-02-15

**Authors:** Laura Choteau, Hélène Vancraeyneste, Didier Le Roy, Laurent Dubuquoy, Luiginia Romani, Thierry Jouault, Daniel Poulain, Boualem Sendid, Thierry Calandra, Thierry Roger, Samir Jawhara

**Affiliations:** 1 INSERM U995/2, Université Lille Nord de France, 1 Place Verdun, 59000 Lille, France; 2 U995-LIRIC, Lille Inflammation Research International Center, University Lille2, 59000 Lille, France; 3 Service de Parasitologie Mycologie, Pôle de Biologie Pathologie Génétique, CHU Lille, 59000 Lille, France; 4 Infectious Diseases Service, Department of Medicine, Lausanne University Hospital, Lausanne, Switzerland; 5 Department of Experimental Medicine and Biochemical Sciences, University of Perugia, Perugia, Italy

**Keywords:** TLR1, TLR2, TLR6, *Candida albicans*, *E. coli*, Colitis

## Abstract

**Background:**

Toll-like receptors (TLRs) are the major pattern recognition receptors that mediate sensing of a wide range of microorganisms. TLR2 forms heterodimers with either TLR1 or TLR6, broadening its ligand diversity against pathogens. TLR1, TLR2 and TLR6 have been implicated in the recognition of *Candida albicans*, an opportunistic fungal pathogen that colonizes the gastrointestinal tract. In this study, we explored whether the deficiency in TLR1, TLR2 or TLR6 impacts *C. albicans* colonization and inflammation-associated colonic injury in the dextran sulfate sodium (DSS)-induced colitis in mice.

**Results:**

DSS treatment and *C. albicans* challenge induced greater weight loss, worse clinical signs of inflammation, higher histopathologic scores, and increased mortality rates in TLR1^−/−^ and TLR2^−/−^ mice when compared to TLR6^−/−^ and wild-type mice. The number of *C. albicans* colonies in the stomach, colon and feces was decreased in TLR6^−/−^ mice as compared to TLR2^−/−^, TLR1^−/−^ and wild-type mice. Interestingly, the population of *E. coli* in colonic luminal contents, intestinal permeability to FITC-dextran and cytokine expression were significantly increased in TLR1^−/−^ and TLR2^−/−^ mice, while they were decreased in TLR6^−/−^ mice.

**Conclusion:**

In contrast to TLR6, both TLR1 and TLR2 deficiencies increased intestinal inflammation, and the overgrowth of *C. albicans* and *E. coli* populations in the colitis model, suggesting the involvement of TLR1 and TLR2 in epithelial homeostasis, and a role of TLR6 in increasing intestinal inflammation in response to pathogen-sensing.

**Electronic supplementary material:**

The online version of this article (doi:10.1186/s13099-017-0158-0) contains supplementary material, which is available to authorized users.

## Background

Toll-like receptors (TLRs) are the main family of pattern recognition receptors (PRRs) through which immune and non-immune cells sense pathogen-associated molecular patterns (PAMPs) [1, 2]. Several TLRs are implicated in the recognition of fungal pathogens such as *Candida albicans* [3, 4]. The interaction between TLRs and yeasts during candidiasis stimulates immune cells to generate inflammatory and immunomodulatory mediators that shape the host immune response. Unlike TLR4, TLR2 recognizes both blastoconidia and hyphal forms of *C. albicans* [5]. TLR2 forms heterodimers with either TLR1 or TLR6 which have been implicated in ligand discrimination [6]. TLR2 senses phospholipomannans, which are expressed in the cell wall of *C. albicans* [7]. In addition, TLR2 in combination with galectin-3 also senses β-mannosides [8].

TLRs are expressed not only in myeloid cells and leukocytes, but also in the intestinal epithelium, which contributes to mucosal homeostasis by preventing the penetration of commensal microbiota into the intestine [9, 10]. In an animal model of colitis, TLR2^−/−^ mice developed more severe colonic inflammation than wild-type mice [11]. Moreover, mutations in TLRs, including the *TLR2* gene, have been associated with predisposition to and maintenance of inflammatory bowel disease (IBD) [12–14]. Interestingly, in patients with ulcerative colitis, Pierik et al. [15] observed an association between *TLR1* and *TLR2* gene polymorphisms and pancolitis, and a negative relationship between *TLR6* polymorphisms and pancolitis, suggesting that TLR2 and its co-receptors TLR1 and TLR6 are involved in the initial immune response to pathogens in the development of IBD.

The aim of this study was to determine the impact of TLR1, TLR2, and TLR6 deficiency on inflammatory parameters associated with *C. albicans* colonization and acute colitis induced by DSS by comparing wild-type, TLR1^−/−^, TLR2^−/−^, and TLR6^−/−^ mice. We also assessed intestinal permeability, serological response, and colonic expression levels of pro-inflammatory and anti-inflammatory cytokines in control and TLR-deficient mice. Finally, we explored the effects of TLR deficiency on neutrophil-mediated *C. albicans* phagocytosis/death.

## Results

### *Candida albicans* CFU in stools and mouse body weight

**Fig. 1:**
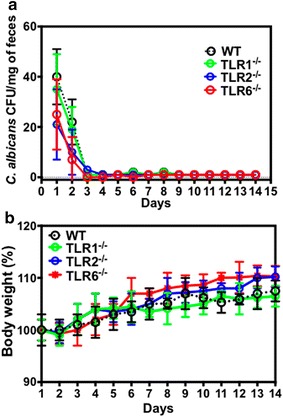
*Candida albicans* colonization and body weight in mice challenged with *C. albicans*. **a** Number of *C. albicans* colony forming units (CFU) recovered from stools. A single inoculum of 10^7^ *C. albicans* was administered to mice on day 1. A total of 40 mice were divided into four groups composed of wild-type Candida (WT, n = 10), TLR1^−/−^ Candida (n = 10), TLR2^−/−^ Candida (n = 10), and TLR6^−/−^ Candida (n = 10). Data are mean ± SE of two independent experiments. **b** Mouse body weight. There were no differences between wild-type and TLR deficient mice in terms of body weight changes. Data are mean ± SE of two independent experiments

TLR1^−/−^, TLR2^−/−^, TLR6^−/−^, and wild-type mice were challenged with a single oral inoculum of *C. albicans* (10^7^ CFU) and the amount of yeast in stool samples was analyzed daily for 2 weeks to assess the colonization rate (Fig. 1a). *C. albicans* colonization was not observed in any of these mice a few days later. In the absence of DSS, no significant differences in body weight were observed between TLR deficient mice and wild-type mice that received *C. albicans* (Fig. 1b). Additionally, there were no differences between wild-type and TLR deficient mice that challenged with *C. albicans* in terms of clinical and histological scores (data not shown).

### Mouse weight and survival analysis in DSS-induced murine colitis

**Fig. 2:**
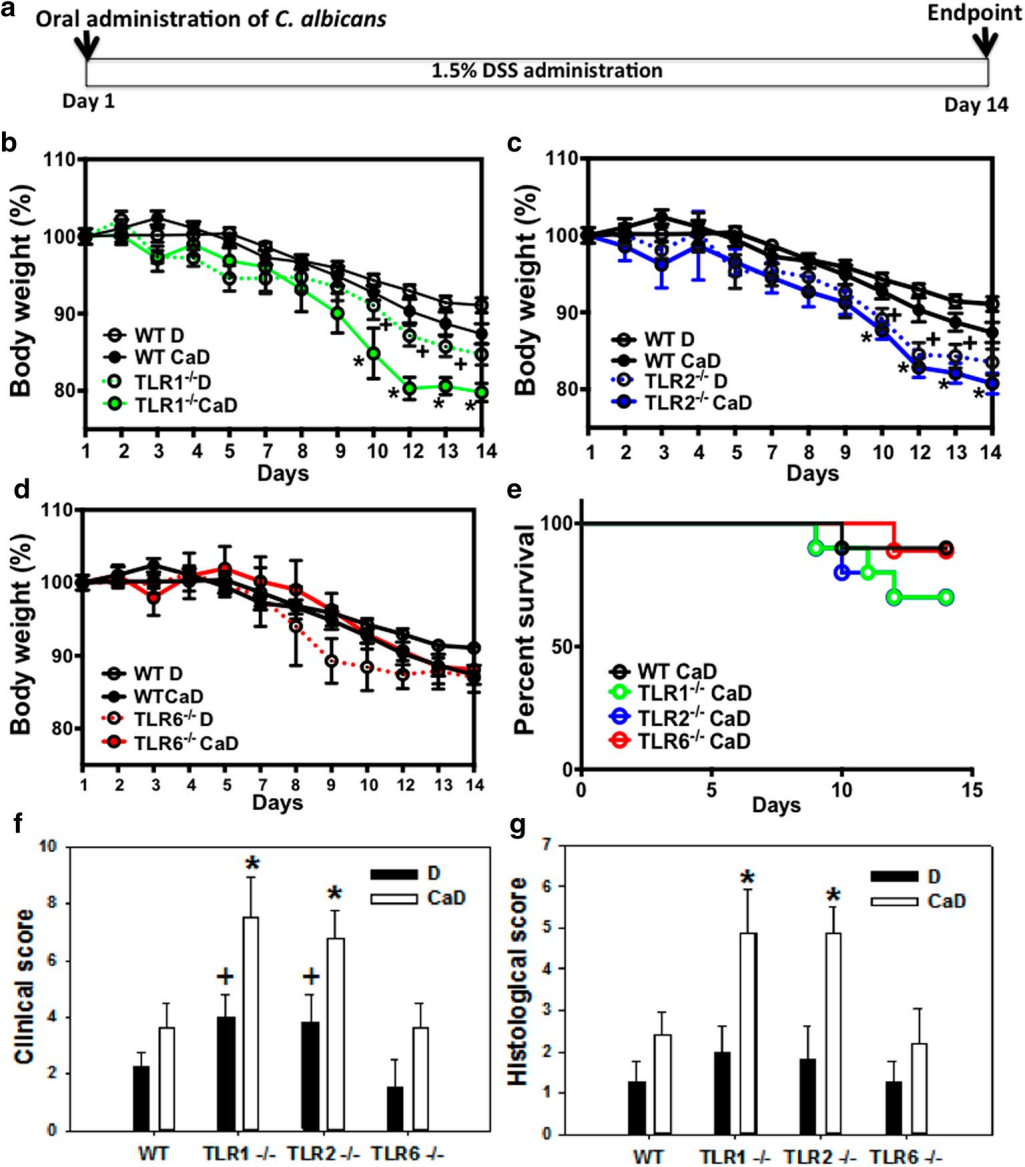
Increased morbidity and mortality of TLR1^−/−^ and TLR2^−/−^ mice due to *C. albicans* and DSS-induced colitis. **a** Schematic representation of the experimental procedure. A single inoculum of 10^7^ *C. albicans* was administered to mice on day 1 and low doses of DSS (1.5%) were given in the drinking water for 2 weeks. A total of 120 mice were divided into eight groups composed of wild-type DSS (WT D, n = 10), wild-type *C. albicans* and DSS (WT CaD, n = 20), TLR1^−/−^ D (n = 10), TLR1^−/−^ CaD (n = 20), TLR2^−/−^ D (n = 10), TLR2^−/−^ CaD (n = 20), TLR6^−/−^ D (n = 10), and TLR6^−/−^ CaD (n = 20) mice. **b**–**d** Mouse body weight. Data are the mean ± SE of two independent experiments. ^+^ *P*<0.05 for TLR-deficient CaD mice versus wild-type D mice. *P < 0.05 for TLR-deficient CaD mice versus wild-type CaD mice. **e** Mouse survival. Results are expressed as percent survival from the time of *C. albicans* challenge and DSS treatment. The survival data were significantly different by the log-rank test (*P* < 0.05). **f** Clinical analysis of DSS-induced colitis in mice. Clinical score was determined by assessing weight loss, change in stool consistency and presence of gross bleeding. The clinical score ranged from 0 to 8 (each value corresponds to the mean value of 14 days per group). ^+^ *P*<0.05 for TLR1^−/−^ DSS (**d**) and TLR2^−/−^ D mice versus wild-type (WT) D mice; and *P < 0.05 for TLR1^−/−^ *C. albicans* and DSS (CaD) and TLR2^−/−^ CaD mice versus wild-type CaD mice. **g** Histologic scores. Mice were exposed to 1.5% DSS in drinking water for 14 days. Scores range from 0 (no changes) to 6 (extensive cell infiltration and tissue damage). *P < 0.05 for TLR1^−/−^ CaD and TLR2^−/−^ CaD mice versus wild-type CaD mice

To assess the association between TLR1, TLR2 or TLR6 deficiency and *C. albicans* colonization in DSS-induced murine colitis, mice were monitored daily for 2 weeks for body weight loss and survival after a single oral challenge with *C. albicans* and DSS treatment (Fig. 2a). All mice treated with DSS showed significant body weight loss, and no mortality was observed. Interestingly, *C. albicans* colonization caused a greater body weight loss in TLR1^−/−^ and TLR2^−/−^ DSS mice when compared to TLR6^−/−^ and wild-type DSS-treated mice (Fig. 2b–d). From day 9, when compared to wild-type mice treated with *C. albicans* and DSS, there was a significant decrease in body weight of TLR1^−/−^ and TLR2^−/−^ mice treated with *C. albicans* and DSS. Furthermore, the *C. albicans* and DSS-treated mouse survival rate was lower in TLR1^−/−^ and TLR2^−/−^ mice (86% survival) than in TLR6^−/−^ and wild-type mice (93% survival) (Fig. 2e).

### Clinical and histologic inflammation scores

**Fig. 3:**
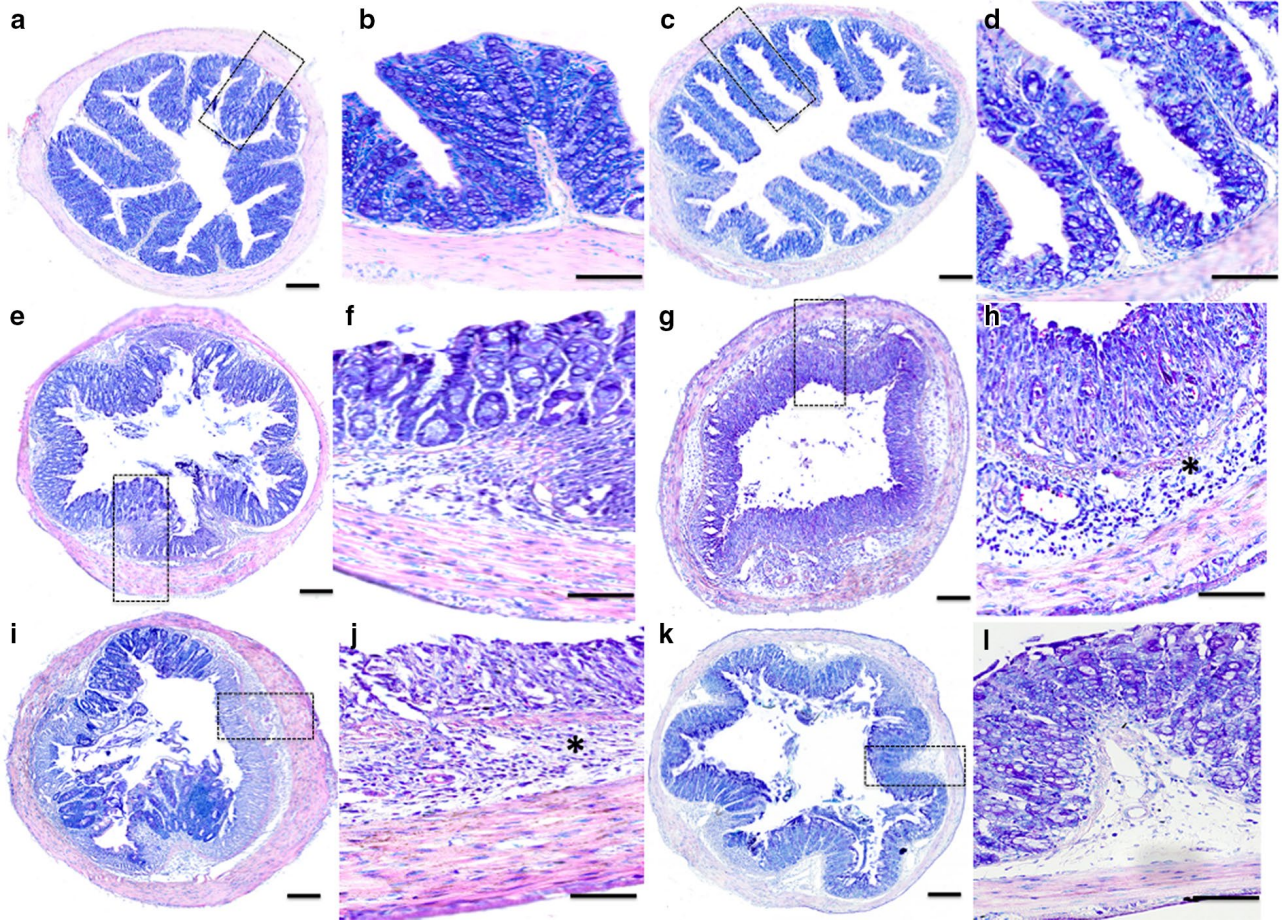
Histologic analysis of the colon in *C. albicans* and DSS-induced colitis. **a**, **c** correspond to colon sections from wild-type mice receiving water as controls (CTL), and *C. albicans* (Ca) respectively. **e**, **g**, **i**, and **k** correspond to colon sections from wild-type *C. albicans* and DSS (CaD), TLR1^−/−^ CaD, TLR2^−/−^ CaD, and TLR6^−/−^ CaD mice, respectively. In the absence of DSS, no significant differences in the colon sections were observed between control animals (not inoculated) and those that received *C. albicans* (**a**, **c**). The colon sections from wild-type CaD and TLR6^−/−^ CaD mice show an inflammatory cell infiltrate in colonic wall structures (**b**, **h**). The colon sections from TLR1^−/−^ CaD and TLR2^−/−^ CaD mice display a high inflammatory cell infiltrate in colonic wall structures and massive tissue destruction (*asterisk*, **h**, **j**). The *scale bars* represent 50 µm (**a**, **c**, **e**, **g**, **i**, **k**) and 10 µm (**b**, **d**, **f**, **h**, **j l**)

To evaluate clinical inflammation scores, mice were monitored daily using the parameters of stool consistency and the presence or absence of faecal blood (Fig. 2f). In the absence of *C. albicans* challenge, no significant difference in clinical scores for inflammation was observed between DSS-treated TLR6^−/−^ and DSS-treated wild-type mice. In contrast, clinical scores for inflammation were significantly higher in TLR1^−/−^ and TLR2^−/−^ mice than in wild-type DSS-treated mice. Importantly, the clinical symptoms of colitis, such as diarrhea and bloody stools, appeared more rapidly in TLR1^−/−^ and TLR2^−/−^ mice than in wild-type and TLR6^−/−^ *C. albicans* and DSS-treated mice. Microscopic inflammatory changes were also assessed in mice colons (Fig. 2g). All surviving animals were sacrificed on day 14 and histologic injury scoring was performed on the colons. Histologic scores, based on the extent of infiltration of inflammatory cells and the degree of tissue damage, ranged from 0 to 6, and reflected inflammation and crypt damage caused by DSS. No significant difference in the histologic score was detected between wild-type and TLR-deficient DSS-treated mice. In contrast, histologic scores were substantially higher in *C. albicans* and DSS-treated TLR1^−/−^ and TLR2^−/−^ as compared to TLR6^−/−^ and wild-type mice (*P* < 0.05). Epithelial damage occurred throughout the colonic mucosa, and the infiltrated cells were mostly polynuclear (Fig. 3). In addition, cryptic abscesses and mucosal edema was more frequently observed in the colons of TLR1^−/−^ and TLR2^−/−^ mice than in those of TLR6^−/−^ and wild-type *C. albicans* and DSS-treated mice (Fig. 3).

### Effects of DSS-induced colitis on *C. albicans* colonization

**Fig. 4:**
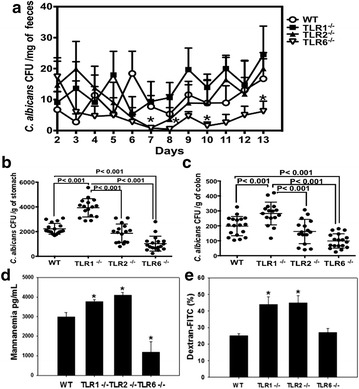
*Candida albicans* colonization in mouse DSS-induced colitis. **a** Number of *C. albicans* colony forming units (CFU) recovered from stools. Data are the mean ± SD of 20 mice per group. *P < 0.05 for TLR6^−/−^ *C. albicans* and DSS (CaD) mice versus wild-type (WT) CaD mice. **b**, **c** Number of *C. albicans* CFU recovered from the stomach and colon. Data are the mean ± SD of 20 mice per group (*P* < 0.001). **d** Mannan levels in the serum of wild-type, TLR1^−/−^, TLR2^−/−^, and TLR6^−/−^ CaD mice. *P < 0.05 for TLR1^−/−^ CaD, TLR2^−/−^ CaDSS and TLR6^−/−^ CaD mice versus wild-type CaDSS mice. **e** FITC-dextran permeability in the colons of mice. Mice were given FITC-dextran by gavage (500 mg/kg body weight). After 4 h, blood samples were collected and fluorescence levels were measured. *P < 0.05 for TLR1^−/−^ CaD, and TLR2^−/−^ CaD mice versus wild-type CaD mice

Mice were challenged with a single oral inoculum of *C. albicans* (10^7^ CFU) and given 1.5% DSS in drinking water for 14 days (Fig. 4a). Despite a trend towards higher number of *C. albicans* CFUs in the stools of TLR1^−/−^ mice, there was no significant difference between wild-type, TLR1^−/−^, and TLR2^−/−^ mice during the entire observation period. In contrast, the number of *C. albicans* CFUs strongly decreased in the stools of TLR6^−/−^ mice on day 7 after challenge, and remained significantly lower than that of wild-type mice up to day 13 (*P* < 0.05).

To evaluate *C. albicans* colonization in the gut, the number of yeasts adhering to the stomach and colon was determined (Fig. 4b, c). Significantly higher numbers of CFUs were observed in the stomach of TLR1^−/−^ mice when compared to TLR2^−/−^, TLR6^−/−^, and wild-type mice (*P* < 0.001). *C. albicans* did not disseminate to the lungs, heart, liver, spleen or kidneys of wild-type and TLR deficient mice (data not shown).

### Serologic analysis and intestinal permeability

To evaluate the association between anti-mannan antibody titers, *C. albicans* colonization, and TLR deficiency in DSS-treated mice, we assessed mannanemia, which correlates to mannan levels in the blood (Fig. 4d). Mannanemia was higher in TLR1^−/−^ and TLR2^−/−^ mice as compared to wild-type *C. albicans* and DSS-treated mice (*P* < 0.05), which correlated with the higher clinical and histologic scores for inflammation in TLR1^−/−^ and TLR2^−/−^ mice. Additionally, mannanemia was significantly lower in TLR6^−/−^ than that of wild-type mice (*P* < 0.05). Next, we assessed the in vivo intestinal permeability using fluorescein isothiocyanate (FITC)-dextran (Fig. 4e). Intestinal permeability of FITC-dextran was significantly increased in TLR1^−/−^ and TLR2^−/−^ as compared to TLR6^−/−^ and wild-type *C. albicans* and DSS-treated mice (*P* < 0.05). A damaged intestinal barrier in TLR1^−/−^ and TLR2^−/−^ mice following *C. albicans* colonization and DSS treatment is also consistent with the clinical symptoms and severe epithelial damage in the colons of these mice.

### Effects of DSS-induced colitis and *C. albicans* colonization on the growth of the *E. coli* population

**Fig. 5:**
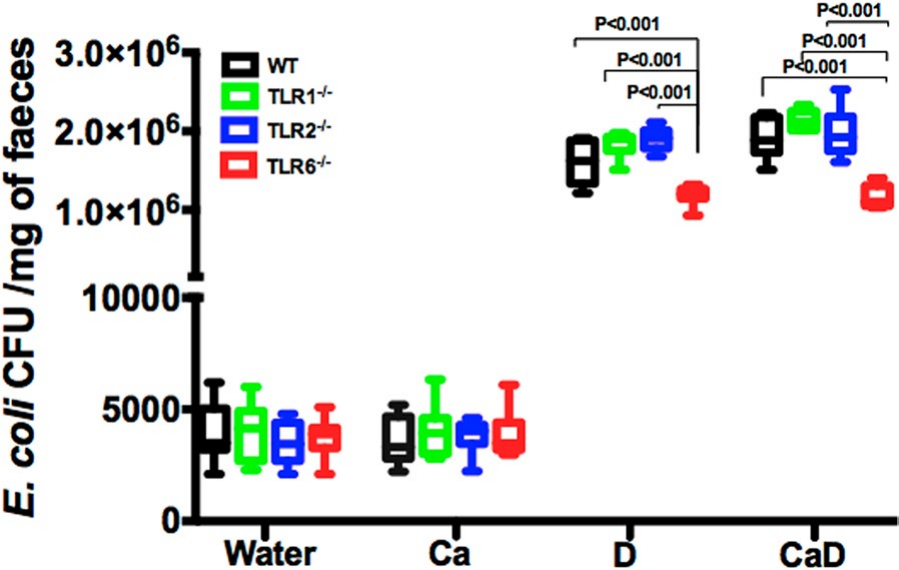
*Escherichia coli* overgrowth in DSS-induced colitis. Four groups composed of controls (water), *C. albicans* alone (Ca), DSS alone (D), and *C. albicans* + DSS (CaD). Data are the mean ± SD of 10 mice per group (*P* < 0.001 for TLR1^−/−^, TLR2^−/−^, or wild-type mice versus TLR6^−/−^ mice)

To assess the impact of DSS-induced colitis and *C. albicans* colonization on the growth of the *E. coli* population, the colonic luminal contents were analyzed at day 14 in all groups. We found that *C. albicans* alone did not induce any changes in the *E. coli* population. However, DSS-induced colitis promoted an increased *E. coli* population in TLR1^−/−^, TLR2^−/−^ and wild-type mice, but a decrease in TLR6^−/−^ mice, regardless of *C. albicans* colonization (Fig. 5).

### Colonic expression levels of cytokines

**Fig. 6:**
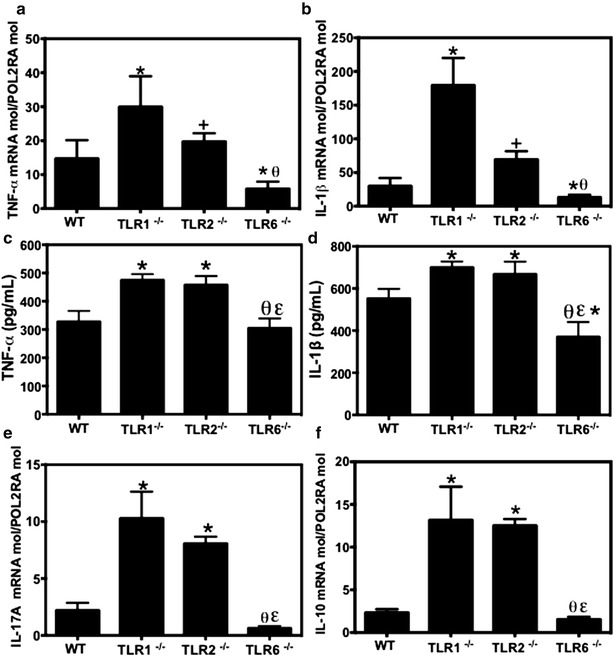
Cytokine expression in *C. albicans* and DSS-induced colitis. **a**, **b** Relative expression levels of TNF, and IL-1β mRNA in mouse colons. **c**, **d** Protein levels of TNF, and IL-1β in mouse colons. **e**, **f** Relative expression levels of IL-17, and IL-10 mRNA in mouse colons. Data are the mean ± SE of 20 mice per group (*P* < 0.05). *P < 0.05 for TLR1^−/−^, TLR2^−/−^, or TLR6^−/−^ CaD mice versus wild-type (WT) CaD mice. ^θ^ *P* < 0.05 for TLR6^−/−^ CaD mice versus TLR1^−/−^ CaD mice, ^ε^ *P* < 0.05 for TLR6^−/−^ CaD mice versus TLR2^−/−^ CaD mice, ^+^ *P*<0.05 for TLR2^−/−^ CaD mice versus TLR1^−/−^ CaD mice

IL-1β and TNF are major pro-inflammatory cytokines that are rapidly released after tissue injury and fungal infection, so we determined their expression levels in the colons of mice challenged with *C. albicans* and treated with DSS (Fig. 6a–d). Both the mRNA expression levels and protein levels of IL-1β and TNF were significantly increased in the colons of TLR1^−/−^ and TLR2^−/−^ mice, whereas their expression levels were significantly reduced in TLR6^−/−^ mice when compared to wild-type mice (*P* < 0.05). Interestingly, both TLR1^−/−^ and TLR2^−/−^ mice had higher expression levels of IL-10 and IL-17A when compared to TLR6^−/−^ and wild-type mice (Figs. 5f, 6e, *P* < 0.05).

### Impact of TLR1, TLR2, or TLR6 deficiency on neutrophil-mediated *C. albicans* phagocytosis and death

**Fig. 7:**
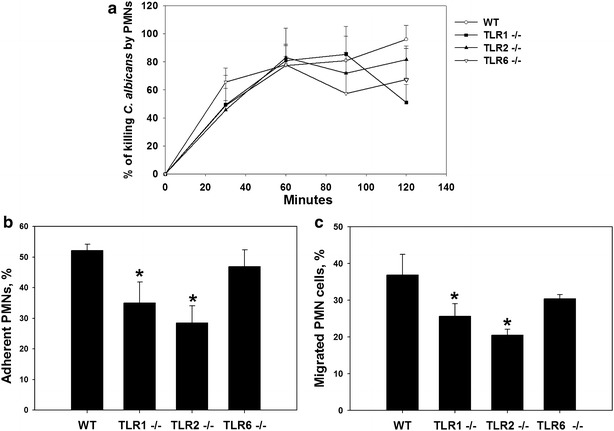
Cell death, adhesion, and migration of neutrophils against *C. albicans*. **a** Cell death assay. 10^5^ *C. albicans* cells were suspended in RPMI and mixed with 5 × 10^5^ neutrophils. *C. albicans* was counted every 30 min. Results are expressed as percent death. **b** Adhesion of neutrophils to *C. albicans. C. albicans* yeasts were incubated for 1 h with neutrophils. Results are expressed as percent neutrophils adhering to yeasts. *P < 0.05 for TLR1^−/−^, and TLR2^−/−^ neutrophils versus wild-type (WT) neutrophils. **c** Migration of neutrophils towards *C. albicans.* The migration of neutrophils towards *C. albicans* was performed using a transwell assay. Results are expressed as the percent neutrophils migrating from the upper to the lower chamber. Data are the mean ± SE of three independent experiments (*P* < 0.05). *P < 0.05 for TLR1^−/−^, and TLR2^−/−^ neutrophils versus wild-type neutrophils

Neutrophils are essential phagocytic cells involved in antifungal immunity. Therefore, we assessed the phagocytic activity of thioglycolate-elicited neutrophils from TLR1^−/−^, TLR2^−/−^, TLR6^−/−^, and wild-type mice (Fig. 7a). Neutrophils and germinated *C. albicans* were co-incubated for 2 h, and the number of viable fungi was quantified every 30 min. We did not find any significant differences in *C. albicans* death. Additionally, live cell video microscopy showed no differences in the engulfment of *C. albicans* by TLR1^−/−^, TLR2^−/−^, TLR6^−/−^, and wild-type neutrophils (data not shown). To examine whether neutrophils utilize TLR1, TLR2, or TLR6 for adhesion to *C. albicans*, we quantified the CFUs of *C. albicans* after 1 h of co-incubation with neutrophils (Fig. 7b). Neutrophils from TLR1^−/−^ and TLR2^−/−^ mice showed significantly less adherence to *C. albicans* than those from TLR6^−/−^ and wild-type mice (*P* < 0.05). We also assessed the migration of neutrophils towards germinated *C. albicans* cells after 8 h of co-incubation using a transwell assay (Fig. 7c). We observed less TLR1^−/−^ and TLR2^−/−^ neutrophil migration towards *C. albicans* than TLR6^−/−^ and wild-type neutrophil migration (*P* < 0.05).

## Discussion

IBD is characterized by microbial dysbiosis related to the abundance of a range of pathogenic microbial species, in particular, *C. albicans* [16–18]. *C. albicans* colonization is more frequent and more severe in patients with Crohn’s disease than in control subjects [19]. The abundance of yeast in the gut can cause inflammation by activating cells through PRRs, including TLRs [20, 21]. TLRs are highly expressed in mucosal immune and epithelial cells and their triggering stimulates cytokine release and microbicidal activity [22]. In the present study, we investigated the role of TLR1, TLR2, and TLR6 in intestinal inflammation and *C. albicans* colonization. We previously developed a *C. albicans* colonization model, which is promoted by intestinal inflammation induced by DSS [23]. This *C. albicans* colonization is not maintained without the presence of intestinal inflammation; otherwise *C. albicans* is eliminated immediately in mice. In this DSS model, we employed a low concentration of DSS (1.5%) to promote *C. albicans* colonization without inducing mouse mortality or severe colitis. In the present study, we compared DSS mice and mice treated with DSS and colonized with *C. albicans*. Initially, we performed experiments without DSS by administering *C. albicans* only to mice used as controls (TLR1^−/−^, TLR2^−/−^, TLR6^−/−^, and wild-type mice). No *C. albicans* colonization was observed in any of these mouse strains a few days later. Interestingly, we observed that TLR1^−/−^ and TLR2^−/−^ mice were more susceptible to DSS-induced colitis than TLR6^−/−^ and wild-type mice. Rakoff-Nahoum et al. [10] demonstrated that mice deficient in TLR2 are highly susceptible to DSS-induced colitis, suggesting that TLRs contribute to prevent an aberrant immune response against the commensal flora. TLR2 forms heterodimers with either TLR1 or TLR6 to recognize different configurations of lipoproteins/lipopeptides [6]. It has been reported that TLR2/TLR6 recognizes yeast-derived zymosan, which is mainly composed of mannans and β-glucans [24]. Jouault et al. [7, 8] showed that TLR2 binds to phospholipomannans of *C. albicans* in the presence of galectin-3, and β-mannosides of *C. albicans*, to induce pro-inflammatory responses by macrophages. TLR2 can also collaborate with dectin-1 to bind fungal β-glucans [25, 26].

We observed that *C. albicans* colonization enhances the susceptibility of TLR1^−/−^ and TLR2^−/−^ mice, but not TLR6^−/−^ mice, to intestinal inflammation. TLR6^−/−^ mice were more resistant to DSS-induced colitis as reflected by lower clinical and histologic scores for inflammation and mortality. These data corroborate findings from a recent study which showed that mice deficient in TLR6^−/−^ are protected from intestinal inflammation and have a low number of Th17 cells [27]. This evidence emphasizes the role played by TLR6 in the induction of an inflammatory response to *C. albicans* sensing. Netea et al. [28] showed that, in contrast to TLR1, TLR6 is involved in the recognition of *C. albicans* and modulation of the Th1/Th2 cytokine balance. Moreover, TLR2^−/−^ mice had significantly impaired survival to *Candida* infection, indicating that TLR2 confers protection against primary disseminated candidiasis [3].

All TLRs except TLR3 are expressed by neutrophils, which represent the primary first line of defense against *C. albicans* infection [1, 29]. In the present study, neutrophils from TLR1, TLR2, and TLR6 deficient mice were not affected in terms of *C. albicans* phagocytosis. On the other hand, adhesion and migration of neutrophils from TLR1^−/−^ and TLR2^−/−^ mice to *C. albicans* was impaired. In line with these findings, neutrophils and macrophages from TLR2^−/−^ mice internalized and killed *C. albicans* as efficiently as wild-type cells [4]. Moreover, Weindl et al. [30] showed neutrophil-dependent TLR4-mediated protective mechanisms against *C. albicans* infection at epithelial surfaces, a phenomenon that was independent of neutrophil migration to *C. albicans* or epithelial cells.

Analysis of colon cytokine expression revealed that TLR1 deficiency strongly up-regulated TNF, IL-1β, and IL17A, whereas TLR6 deletion down-regulated TNF, IL-1β, and IL17A colonic expression when compared to controls. These data suggest that TLR1 plays a role in dampening the cytokine response and preventing excessive immune-mediated tissue damage, whereas TLR6 is involved in exacerbating the inflammatory response associated with tissue damage upon recognition of *C. albicans* and DSS-induced colitis [31, 32]. Notably, *C. albicans* colonization increased TLR2 expression in the colons of DSS-treated wild-type mice, whereas TLR2 expression was reduced in galectin-3 deficient mice leading to high pro-inflammatory cytokine expression and aggravated intestinal inflammation [20].

IL-10 is essential for host defense against *C. albicans* infection and can limit the potential tissue damage caused by inflammation [33]. In the present study, we showed that in contrast to the state of TLR6^−/−^ mice, DSS-induced colitis induced the production of pro- and anti-inflammatory cytokines, including IL-10, in TLR1^−/−^ mice. In line with this study, the absence of TLR6 dramatically increased survival and decreased IL-10 production, whereas the absence of TLR1 led to decreased survival and higher IL-10 levels in mice infected with *Yersinia pestis*, suggesting that TLR6 is a distinct TLR receptor driving regulatory IL-10 responses [34]. Netea et al. [35] showed that TLR activation, in particular TLR2, can suppress the immune defense against *C. albicans* through the induction of IL-10 and regulatory T-cells.

In this study, we observed that TLR6^−/−^ mice eliminate *C. albicans* from the digestive tract more rapidly than TLR1^−/−^ and TLR2^−/−^ mice. In particular, colonization in the stomach was dramatically higher in TLR1^−/−^ mice, supporting the notion of a protective role of TLR1 and TLR2 against *C. albicans* colonization. Additionally, in agreement with our results, *TLR1* gene polymorphisms have been associated with an increased susceptibility to candidemia in patients [21]. In addition, polymorphisms in the *TLR2* gene have been identified as a risk factor for candidemia [36, 37]. Although TLR2 interacts with a large number of non-TLR molecules, in particular Galectin-3 and Dectin-1-mediated ERK/MAPK activation [8, 25], allowing the recognition of fungal ligand varieties, in our study, we found that mice deficient in TLR1 had higher levels of colonization with *C. albicans* in the stomach and colon than TLR2^−/−^ mice. These data show that TLR1 is more involved in *C. albicans* elimination from the gut than TLR2, suggesting that the absence of TLR1 can have a great impact on the formation of the heterodimer TLR1/TLR2 when compared to the absence of TLR2. Van Duin et al. [38] showed that alterations in baseline TLR1 surface expression were increased in elderly individuals, whereas TLR2 surface expression was unaffected, suggesting that the defect in TLR1 expression can contribute to the increased infection-related morbidity and mortality observed in elderly individuals.

Changes in commensal bacterial diversity can differentially modulate mucosal TLR responsiveness, leading to TLR-mediated hyper- or hypo-reactive immune responses [10, 39, 40]. In the present study, intestinal inflammation alone was sufficient to promote the overgrowth of *E. coli*. Additionally, both inflammation and *C. albicans* colonization maintained *E. coli* overgrowth in TLR1^−/−^, TLR2^−/−^ and wild-type mice when compared to that in TLR6^−/−^ mice. These data are consistent with Lupp et al. [41] finding which suggested that the commensal *E. coli* increased during colitis and displayed a strong pro-inflammatory potential. Ey et al. [14] showed that an unaltered microbiota was required for colitis exacerbation in TLR2/MDR1A double-knockout mice once protection from colitis was observed upon antibiotic treatment.

In the present study, intestinal inflammation alone was sufficient to promote the overgrowth of *E. coli*. Additionally, both inflammation and *C. albicans* colonization maintained *E. coli* overgrowth in TLR1^−/−^, TLR2^−/−^ and wild-type mice when compared to that in TLR6^−/−^ mice. These data are consistent with Lupp et al. [41] finding which suggested that the commensal *E. coli* increased during colitis and displayed a strong pro-inflammatory potential.

Experimental and clinical studies emphasize the role of the serologic response during the development of IBD [20, 42]. Crohn’s disease patients who express high levels of serologic markers experience more aggressive disease, suggesting that the presence of Crohn’s disease marker antibodies reflect a specific mucosal immune-mediated response [43]. We observed that mannanemia was elevated only in TLR1^−/−^ and TLR2^−/−^ mice. High mannanemia in TLR1^−/−^ and TLR2^−/−^ mice was correlated with increased intestinal permeability, which facilitates the passage of fungi into the bloodstream. Clinically, Pierik et al. [15] showed a negative association between *TLR6* S249P and ulcerative colitis with proctitis. In our study, we found in a clinical cohort of 26 healthy subjects (between 25- and 65-years-old) an association between the *TLR6* rs5743810 homozygous wild-type genotype and ASCA (anti-*Saccharomyces cerevisiae* antibody) level (*P*  =  0.0317). Homozygous healthy subjects (*TLR6* A/A wild-type) have significantly higher ASCA levels than heterozygous (*TLR6* A/G) and homozygous mutants (*TLR6* G/G) indicating a possible association of this *TLR6* rs5743810 polymorphism with IBD (Additional file 1). These clinical data corroborate findings from our experimental study, which showed that deletion of TLR6 decreased the circulating mannan level in mice. Taylor et al. [44] showed that among African Americans, women who carried 1 or 2 of the *TLR6* rs5743810 alleles had decreased odds of endometritis and upper genital tract infection and there was a similar trend among white participants. We intend to widen our study to larger groups of healthy subjects and patients with CD.

## Conclusions

DSS-induced colitis promoted the overgrowth of *E. coli* and *C. albicans* in the gut. TLR1 and TLR6 had not the same effects on the TLR2-mediated immune response. Deletion of TLR1 exacerbated intestinal inflammation in response to *C. albicans* colonization, resulting in colonic injury and mouse mortality. Conversely, deletion of TLR6 impacted on the intestinal inflammation via the modulation of cytokine expressions and promoted the elimination of *C. albicans*. Overall, this study emphasizes the role of TLRs in the modulation of intestinal inflammation and *C. albicans* colonization and shows the involvement of TLR1 in the homeostasis of intestinal epithelium and the impact of TLR6 on both intestinal inflammation and the host defense.

## Methods

### Yeast strain

*Candida albicans* Sc5314 strain was used throughout this study [45]. *C. albicans* isolates were grown in Sabouraud’s dextrose broth at 37 °C in a shaking incubator for 18 h.

### Animals

Female C57BL/6 mice (8–10-weeks-old) were purchased from Charles River Laboratories (France). All mice were fed with a standard chow diet (Scientific Animal Food and Engineering, SAFE diet-A04, France). This diet contained neither yeast cells nor yeast cell extract. The strain description and use of TLR1^−/−^, TLR2^−/−^, and TLR6^−/−^ C57BL/6 mice has been provided elsewhere [46]. WT and TLRs^−/−^ mice were each distributes into control group and experimental groups and two sets of experiments (a total of 90 mice/experiment) were performed independently. A group of healthy mice was used as controls (CTL, n = 5 WT and n = 15 TLR deficient mice). A second group of mice was gavaged orally with *C. albicans* without any other treatment (Ca, n = 10 WT and n = 30 TLR deficient mice). A third group was treated with DSS alone (D, n = 10 WT and n = 30 TLR deficient mice). A fourth group was treated with DSS and gavaged orally with *C. albicans* (CaD, n = 20 WT and n = 60 TLR deficient mice). Mouse survival and body weight were monitored daily for 14 days. All efforts were made to minimize animal suffering. Mice with a body weight loss of >20% initial body weight were humanely euthanized by inhalation of 5% isoflurane followed by cervical dislocation.

### Inoculum preparation and induction of colitis

Each animal was inoculated on day 1 by single oral gavage with 200 µL of phosphate-buffered saline (PBS) containing 10^7^ *C. albicans* cells. Mice were then given 1.5% DSS (MW 36–50 kDa; MP Biomedicals, LLC, Germany) in drinking water from day 1 to day 14, to induce intestinal inflammation. The presence of yeasts in the intestinal tract was assessed daily by performing plate counts from feces (approximately 0.1 g/sample) [20]. Stool samples were daily collected from each tagged animal and suspended in 1 mL PBS, and plated onto Candi-Select medium (Bio-Rad, Marnes la Coquette, France). The colonies were counted after 48 h incubation at 37 °C. The results were noted as colony forming units (CFU)/µg of feces. To assess *C. albicans* colonization of the gut, the gastrointestinal tract was removed from sacrificed mice, and the stomach and colon were separated and analyzed. The tissues were cut longitudinally. After removal of intestinal contents, the tissues were washed several times with PBS to minimize surface contamination from organisms present in the lumen [47]. Serial dilutions of homogenates were plated onto Sabouraud’s agar plates, and the results were expressed as *C. albicans* CFU/mg of tissue. For the *E. coli* identification, the colonic luminal faecal samples at 14 days were collected from each tagged animal and plated onto MacConkey agar (Sigma-Aldrich) containing Fluconazole (Fresenius Kabi, 60 mg/L) to suppress the growth of fungal cells. The plates were incubated at 37 °C and examined at 24 and 48 h later. For the identification of bacteria, colonies were mixed with 1.5 μL of matrix solution (α-cyano-4-hydroxycinnamic acid [HCCA]; Bruker Daltonics) dissolved in 50% acetonitrile, 47.5% water, and 2.5% trifluoroacetic acid and allowed to dry prior to analysis using the MALDI-TOF MS (Microflex-Bruker Daltonics).

### Assessment of clinical parameters

The body weight and mortality of the mice was recorded daily. The data were expressed as mean percent change from initial body weight. Clinical scores ranging from 0 to 8 were calculated as described elsewhere [20, 48].

### Intestinal permeability in vivo

Mice were given DX-4000-FITC (FD4000, Sigma-Aldrich, France) by oral gavage (500 mg/kg body weight) [49]. After 4 h, blood samples were collected and DX-4000-FITC concentrations were quantified with a Mithras^®^ fluorescence spectrophotometer (Berthold technologies, France).

### Histologic score

The rings of the transverse part of the colon were fixed overnight in 4% paraformaldehyde-acid, embedded in paraffin, and histological analysis was performed by staining the cross-sections (4-µm thick) with hematoxylin-eosin (Sigma-Aldrich, France). Histologic scores were evaluated by two independent blinded investigators who observed two sections per mouse at magnifications of ×10 and ×100. The scores were determined as described by Siegmund et al. [48] and the sections were evaluated for the following two subscores: (1) a score for the presence and confluence of inflammatory cells, in the lamina propria and submucosa or transmural extension; and (2) a score for epithelial damage, focal lymphoepithelial lesions, mucosal erosion and/or ulceration and extension to the bowel wall. The two subscores were added together and the combined histologic score ranged from 0 (no changes) to 6 (extensive cell infiltration and tissue damage). Immunohistofluorescence staining of the colons was performed on dewaxed and PBS-rehydrated paraffin-embedded colon sections. The tissue sections were incubated for 30 min at room temperature in 1% bovine serum albumin (BSA) diluted in PBS. Slides were washed with PBS and incubated for 1 h at room temperature with anti-mouse CD281-TLR1 (eBioscience), anti-mouse CD282-TLR2 (eBioscience), and anti-mouse TLR6 (ab37072, Abcam) antibodies, which were diluted 1:100 in PBS. After 3 washes with PBS, the sections were incubated with fluorescein isothiocyanate-conjugated anti-rabbit or anti-rat antibodies (Zymed Laboratories) for 60 min at 37 °C [50]. The sections were then washed with PBS and counterstained with DAPI. The sections were then examined by confocal microscopy (Zeiss LSM710, diode 561 nm DPSS).

### Measurement of mannan antigen in mouse serum

Mannan antigen was measured using a Platelia™ Candida Ag (mannan) kit (Bio-Rad) according to the manufacturer’s instructions. Absorbance was read at 450 nm (reference filter, 620 nm) in a microplate reader (Bio-Rad) after addition of tetramethylbenzydine [20]. Results were expressed as optical density (OD).

### Real-time mRNA quantification and measurement of cytokine levels in colons

Total RNA was isolated from colon samples using a NucleoSpin RNA II kit (Macherey–Nagel) following the manufacturer’s instructions, with 20–50 units of DNase I (RNase-free) at 37 °C for 30 min to avoid contamination with genomic DNA. RNA was quantified with a Nanodrop (Thermo Scientific, Wilmington, DE). Reverse transcription of mRNA was carried out according to the manufacturer’s instructions (Applied Biosystems). Briefly, reverse transcription of mRNA was performed in a final volume of 20 μL, from 1 μg of total RNA, using 50 units of reverse transcriptase (Applied Biosystems) with 200 nmol oligo (dT) 12–18. Polymerase chain reaction (PCR) was then performed on this mixture using the one-step system (Applied Biosystems) with Fast SYBR green (Applied Biosystems). Amplification was carried out in a total volume of 12 μL containing 0.25 μL of each primer (Table 1) and 2.5 μL of cDNA prepared as described above. SYBR green dye intensity was analyzed using step-one software. All results were normalized to the reference gene *POLR2A* [51]. Cytokine concentrations from colons were measured using a commercial ELISA kit according to the manufacturer’s instructions (eBioscience, San Diego, CA).

**Table 1:** Mouse oligonucleotide sequences

Primers	Sequence (5′–3′)
POLR2A S POLR2A AS	CCCACAACCAGCTATCCTCAA GGTGCTGTGGGTACGGATACA
IL-1β S IL-1β AS	AGCTCTCCACCTCAATGGAC AGGCCACAGGTATTTTGTCG
TNF-α S TNF-α AS	CCACCACGCTCTTCTGTCTA GAGGCCATTTGGGAACTTCT
IL-17A S IL-17A AS	GCAAGAGATCCTGGTCCTGA AGCATCTTCTCGACCCTGAA
IL-10 S IL-10 AS	CAGTACAGCCGGGAAGACAATAA CCGCAGCTCTAGGAGCATGT

*S* sense, *AS* antisense

### Adhesion, migration and phagocytosis

Mice were injected intraperitoneally with 500 µL sterile thioglycolate (3% wt/vol). After 6 h, mice were anaesthetized using 5% isoflurane and sacrificed by cervical dislocation. Neutrophils were collected by washing the peritoneal cavity with 5 mL of cold sterile PBS [52]. Neutrophils were plated in RPMI medium supplemented with 10% heat-inactivated fetal bovine serum (Gibco, France) and 1% penicillin–streptomycin, and incubated for 1 h at 37 °C in 5% CO_2_. Non-adherent cells were collected and centrifuged to isolate the neutrophil pellet. Viability and neutrophil counts were determined by the trypan blue exclusion method (Gibco, France).

Neutrophil adhesion assays were performed as described previously [53, 54]. Briefly, 96-well tissue culture plates (Corning) were pre-coated with polyvinylpyrolidone (PVP, Sigma-Aldrich) and washed with PBS. Then, 10^4^ *C. albicans* yeasts in 0.02 mL RPMI were added to each well and incubated overnight at 37 °C to germinate. To assess neutrophil adhesion to *C. albicans*, yeasts were incubated in YNB broth to prevent germination. The supernatant was removed after incubation, and adherent fungi were carefully washed with PBS. Approximately 5 × 10^4^ calcein-labeled neutrophils were added to each well and the plates were then incubated at 37 °C for 1 h. After several washes, the percentage of adherent neutrophils was determined using a Mithras^®^ fluorometer. Neutrophil adhesion to PVP was subtracted from that of the experimental samples. Results were expressed as percent adhesion. A migration assay was performed as described previously [53, 54]. Briefly, 200 μL calcein-labeled washed neutrophils were added to the upper chamber of the transwell devices (Costar Transwell inserts in a 24-well plate format), and 600 μL RPMI with 10^6^ *C. albicans* yeast was added to the lower chamber. Plates were placed in a humidified incubator at 37 °C and 5% CO_2_ for 8 h. To perform the cell death assay, 10^5^ fungal cells were suspended in 0.2 mL RPMI, and mixed with 5 × 10^5^ (1:5 ratio) neutrophils. The neutrophil/fungus mixtures were incubated at 37 °C with slow shaking for 2 h. To determine the extent of cell death/phagocytosis, aliquots of the cell/fungal suspension were taken every 30 min, diluted with PBS, and subsequently plated in serial dilutions on Sabouraud’s dextrose agar plates. The neutrophils were not lysed before plating, and all fungal cells that remained ingested were recorded as “killed.”

### Statistical analysis

 Data were expressed as the mean ± SE of 20 mice in each group. Statistical analysis was performed with Prism 4.0 from GraphPad and XLSTAT. Data were analyzed using the Mann–Whitney U test to compare pairs of groups. The log-rank test was used to analyze mouse survival. Differences were considered significant when the *P* value was <0.05.

## Additional file

**Additional file 1.** Association between *TLR6* rs5743810 homozygous genotype wild-type and ASCA level.
