# Supplementary material for: Role of TLR1, TLR2 and TLR6 in the modulation of intestinal inflammation and Candida albicans elimination
Source: Gut Pathog. 2017 Feb 15;9:9. doi: 10.1186/s13099-017-0158-0 (PMC5310049; doi:10.1186/s13099-017-0158-0)
Supplement: Supplementary file 1 — Additional file 1. Association between TLR6 rs5743810 homozygous genotype wild-type and ASCA level. [file 13099_2017_158_MOESM1_ESM.doc]

**Role of TLR1, TLR2 and TLR6 in the Modulation of Intestinal Inflammation and *Candida albicans* Elimination**

**Laura Choteau, Hélène Vancraeyneste, Didier Le Roy, Laurent Dubuquoy, Luiginia Romani, Thierry Jouault, Daniel Poulain, Boualem Sendid, Thierry Calandra,**

**Thierry Roger, Samir Jawhara**

**Results**

To understand the genetic risk prediction of IBD in a small cohort of healthy adults, we explored the association of genetic variations in *TLR1*, *TLR2*, and *TLR6* genes with ASCA levels. A total of 26 healthy subjects (between 25- and 65-years-old) were genotyped for several single nucleotide polymorphisms (SNPs) and this was correlated with ASCA levels (Fig.1 and Table 1). We found a significant association between the *TLR6* rs5743810 homozygous genotype wild-type and ASCA level (*P* = 0.0317). Taylor et al. showed that among African Americans, women who carried 1 or 2 of the *TLR6* rs5743810 alleles had decreased odds of endometritis and upper genital tract infection and there was a similar trend among white participants [^1^](#_ENREF_1). We did not observe any association between *TLR1* and *TLR2* SNPs and ASCA levels*.* These preliminary results may lead to improved prophylactic treatment of populations at high-risk of IBD and we intend to widen our study to larger groups of healthy subjects and patients with CD.


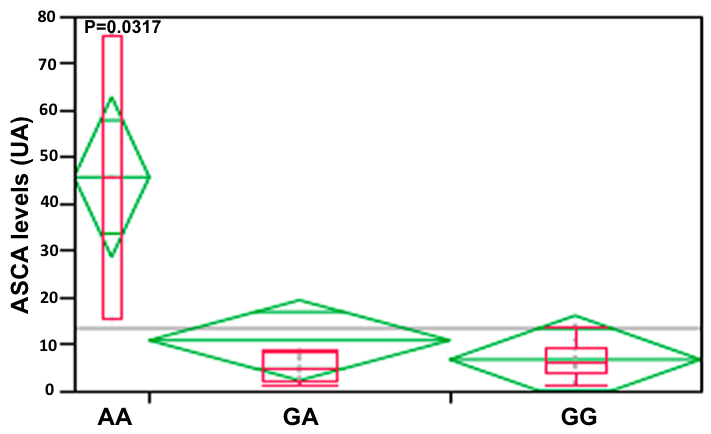


**Figure 1: Association between *TLR6* rs5743810 homozygous genotype wild-type and ASCA level**. Antibody titers (expressed in AU according to the reactivity of the manufacturer’s calibrator) in samples from healthy subjects who were wild-type (A/A), heterozygous (G/A), or homozygous mutants (G/G) for *TLR6* rs5743810. Healthy homozygous subjects (A/A) had higher ASCA levels than those with heterozygous (A/G) and homozygous mutations (G/G).

**Table 1: SNP frequencies (%) in TLR1, TLR2, and TLR6 in a healthy subject.**

| **SNPs** | **Homozygous**  **wild-type** | **Heterozygous** | **Homozygous**  **mutant** | **Allele frequencies** | |
| --- | --- | --- | --- | --- | --- |
| ***TLR1*** |  |  |  | Wild-type | Mutant |
| **rs5743621** | GG: 96.15 | GA: 3.84 |  | G: 99 | A: 1 |
| **rs113706342** | TT: 100 |  |  | T: 99 | G: 1 |
| **rs41311400** | GG: 100 |  |  | G: 99 | A: 1 |
| **rs5743620** | CC: 100 |  |  | C: 99 | T: 1 |
| **rs5743618** | CC: 46.15 | CA: 26.93 | AA: 5 (19.23) | C: 33 | A: 67 |
| **rs5743616** | CC: 100 |  |  |  |  |
| **rs55731057** | CC: 57.69 | CT: 34.62 | TT: 7.7 | C: 48 | T: 52 |
| **rs143576719** | TT: 100 |  |  | T: 96 | C: 4 |
| **rs3923647** | TT: 96.15 | TA: 3.84 |  | T: 98 | A: 2 |
| **rs137873769** | TT: 96.15 | TG: 3.84 |  | T: 100 | G: 0.1 |
| **rs5743611** | CC: 88.46 | CG: 11.53 |  | C: 96 | G: 4 |
| **rs145135062** | TT: 100 |  |  | T:99.8 | C: 0.2 |
| **rs5743610** | GG: 100 |  |  | G: 99 | A: 1 |
| ***TLR2*** |  |  |  |  |  |
| **rs141039581** | CC: 100 |  |  |  |  |
| **rs139227237** | TT: 96.15 | TC: 3.84 |  | T: 99.7 | C: 3 |
| **rs138270311** | GG: 100 |  |  | G: 100 | C: 0.1 |
| **rs3804100** | TT: 92.3 | TC: 7.69 |  | T: 88 | C: 12 |
| **rs5743700** | CC: 96.15 | CT: 3.84 |  | C: 98 | T: 2 |
| **rs5743704** | CC: 96.15 | CA: 3.84 |  | C: 99 | A: 1 |
| ***TLR6*** |  |  |  |  |  |
| **rs5743811** | GG: 100 |  |  | G: 99.9 | A: 0.1 |
| **rs5743810** | AA: 11.53 | GA: 76.93 | GG: 11.54 | A: 19 | G: 81 |

**Materials and methods**

**Ethics statement**

Twenty-six healthy subjects (age range: 25-65 years, 14 women/12 men) were informed about the study and gave their written consent to participate. The study protocol was reviewed and approved by the Ethics Committee of departments of Lille University Hospital (CP 05/86). In addition, the study was conducted according to the principles expressed in the Declaration of Helsinki. No subject had a history of IBD.

**Sequencing analysis**

DNA extractions were performed as described previously [^2^](#_ENREF_2). DNA was extracted using a commercial kit according to the manufacturer’s instructions (Kit Nucleon BACC3, GE Healthcare). For library preparation, AmpliSeq libraries were prepared using the Ion AmpliSeq Library Kit 2.0 and an Ion AmpliSeq custom panel (Life Technologies). Ampliseq technologies were used to design a custom NGS library including 110 amplicons in two pools, covering all our targets of interest (19.87 kb covered at 99.42%). The targets of interest were *TLR1*-ex1, *TLR1*-Ile602Ser, *TLR1*-Asn248Ser, *TLR1*-Arg80>Thr, *TLR1*-7202G/A, *TLR6*-Ser249Pro, and *TLR2* genes. The design is available on request. 10 ng of each DNA sample was used as a template to prepare the library. Quality control of all libraries was performed with an Agilent Bioanalyzer using High Sensitivity chips. Template dilutions were calculated after library concentrations were normalized to ~100 pM using an Ion Library Equalizer kit (Life Technologies). Library templates were clonally amplified using an Ion One Touch 2™, following the manufacturer’s protocol. Recovered template-positive ion sphere particles were subjected to enrichment according to the manufacturer’s instructions. Samples were subjected to the Ion PGM 200 Sequencing v2 protocol using Ion 318 v2 chips (Life Technologies). 32 barcoded samples were loaded per chip to ensure an average depth of 1500. For data analysis, alignment of the sequences to the human genome build 19 reference genome and base calling were performed using Torrent Suite software. Identification of variants was performed with an Ion Torrent Variant Caller and coverage analysis was generated using coverage analysis plugins (Life Technologies). Allelic frequencies and association with ASCA levels were determined with Haploview software [^3^](#_ENREF_3).

**Measurement of anti-mannan antibodies**

Antibodies against *S. cerevisiae* mannan (ASCA) were detected by enzyme linked immunosorbent assay (ELISA) (IBDX gASCA, Glycominds, Isreal) [^4^](#_ENREF_4). Briefly, 50 μL of 1:100 diluted serum was added to the coated wells. Absorbance was read at 450 nm (reference filter, 620 nm) in a microplate reader (Bio-Rad) after addition of tetramethylbenzydine [^5^](#_ENREF_5). Results were expressed as optical density (OD).


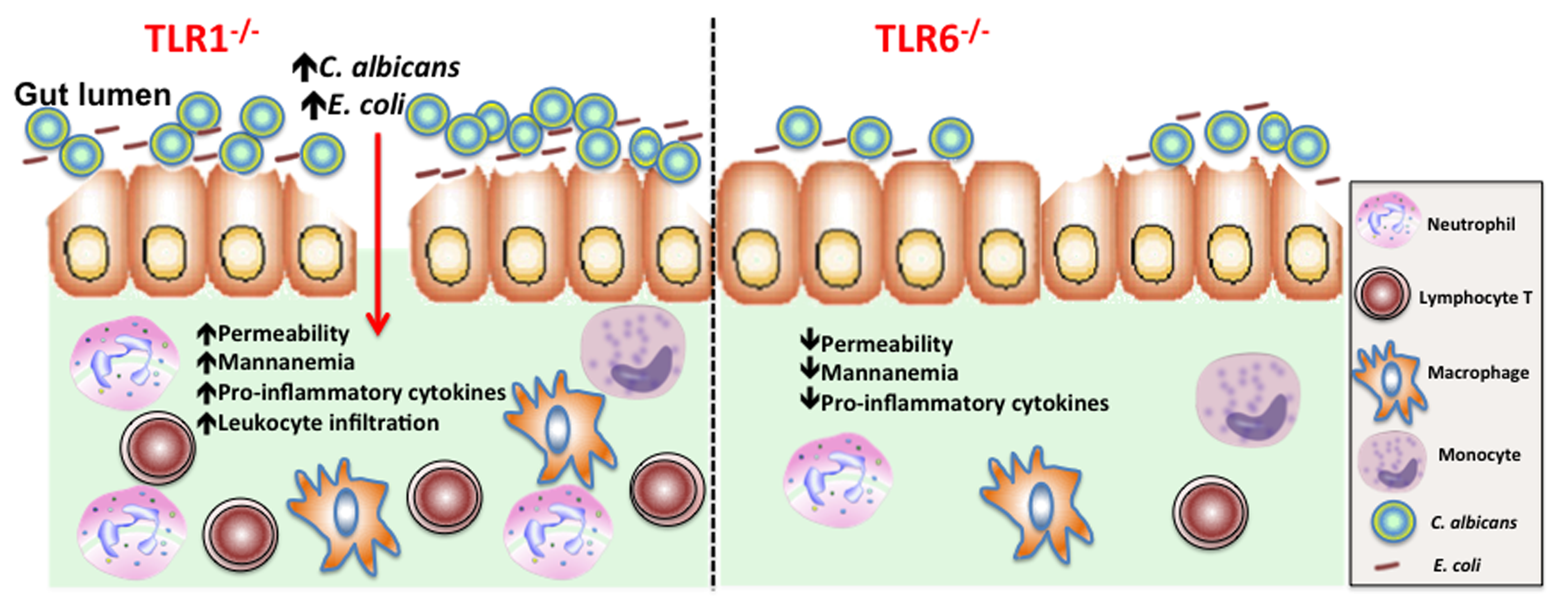


**TLR1 and TLR6 have opposite effects on TLR2-mediated responses in DSS-induced colitis.** TLR1 and TLR6 have opposite effects on TLR2-mediated responses in DSS-induced colitis. Deletion of TLR6 impacted on intestinal inflammation via modulation of cytokine expression, promoted the elimination of *C. albicans* and reduced the intestinal permeability and mannanemia. On the other hand, deletion of TLR1 exacerbated intestinal inflammation in response to *C. albicans* colonization, resulting in colonic injury, *E. coli* overgrowth, high intestinal permeability, leucocyte infiltration and pro-inflammatory cytokine production.

**References**

1 Taylor, B. D., Darville, T., Ferrell, R. E., Ness, R. B. & Haggerty, C. L. Racial variation in toll-like receptor variants among women with pelvic inflammatory disease. *The Journal of infectious diseases* **207**, 940-946, doi:10.1093/infdis/jis922 (2013).

2 Choteau, L. *et al.* Role of mannose-binding lectin in intestinal homeostasis and fungal elimination. *Mucosal immunology*, doi:10.1038/mi.2015.100 (2015).

3 Barrett, J. C., Fry, B., Maller, J. & Daly, M. J. Haploview: analysis and visualization of LD and haplotype maps. *Bioinformatics* **21**, 263-265, doi:10.1093/bioinformatics/bth457 (2005).

4 Sendid, B. *et al.* Antibodies against glucan, chitin, and *Saccharomyces cerevisiae* mannan as new biomarkers of *Candida albicans* infection that complement tests based on *C. albicans* mannan. *Clinical and vaccine immunology : CVI* **15**, 1868-1877, doi:10.1128/CVI.00200-08 (2008).

5 Jawhara, S. *et al.* Colonization of mice by *Candida albicans* is promoted by chemically induced colitis and augments inflammatory responses through galectin-3. *The Journal of infectious diseases* **197**, 972-980, doi:10.1086/528990 (2008).
